# Utilizing machine learning with knockoff filtering to extract significant metabolites in Crohn’s disease with a publicly available untargeted metabolomics dataset

**DOI:** 10.1371/journal.pone.0255240

**Published:** 2021-07-29

**Authors:** Shoaib Bin Masud, Conor Jenkins, Erika Hussey, Seth Elkin-Frankston, Phillip Mach, Elizabeth Dhummakupt, Shuchin Aeron

**Affiliations:** 1 Department of Electrical and Computer Engineering, Tufts University, Medford, MA, United States of America; 2 DEVCOM Soldier Center, Natick, MA, United States of America; 3 DEVCOM Chemical Biological Center, Aberdeen Proving Ground, Aberdeen, MD, United States of America; Universidade Catolica Portuguesa Escola Superior de Biotecnologia, PORTUGAL

## Abstract

Metabolomic data processing pipelines have been improving in recent years, allowing for greater feature extraction and identification. Lately, machine learning and robust statistical techniques to control false discoveries are being incorporated into metabolomic data analysis. In this paper, we introduce one such recently developed technique called aggregate knockoff filtering to untargeted metabolomic analysis. When applied to a publicly available dataset, aggregate knockoff filtering combined with typical p-value filtering improves the number of significantly changing metabolites by 25% when compared to conventional untargeted metabolomic data processing. By using this method, features that would normally not be extracted under standard processing would be brought to researchers’ attention for further analysis.

## Introduction

Inflammatory bowel disease (disease (IBD) is an umbrella term that describes conditions like ulcerative colitis (UC) and Crohn’s disease (CD). IBD is referred to as a symptom cluster, where numerous pathologies result in a subset of symptoms, diagnosis methods, and treatments [1]. This class of disorders is generally characterized by diarrhea, rectal bleeding, abdominal pain, weight loss and fatigue [1]. Ulcerative colitis, CD, and additional disorders, like inflammatory bowel disease unclassified (IBDU) are generally diagnosed and classified on a spectrum of clinical and endoscopic criteria [2]. In recent years, due to the advancement in mass spectrometry in clinical medicine, biomarkers have been proposed to aid in the diagnosis of these inflammatory bowel diseases [3–5]. As a result of IBD is a being classified as a spectrum disorder, and thus there is a desire to observe a correlation associated with regulation of these biomarkers (in either an up or down manner) and severity of disorder.

Important biomarker identification in metabolomics that differentiate two or more groups, has been studied widely using univariate and multivariate statistical feature selection methods. Based on the knowledge of the feature distribution, both parametric as well as non-parametric univariate statistical techniques e.g., ANOVA, Student’s t test, Kolmogorov-Smirnov test, Mann-Whitney U test, Kruskal-Wallis one way analysis of variance test [6–9] have been used to select significant metabolites. These univariate methods perform multiple hypothesis tests (one hypothesis per feature), and an additional correction method is required to adjust for multiple hypothesis testing. A typical correction method, called the Bonferroni correction [10] is very conservative and leads to a lot of false negatives, especially if the number of features is very large. Benjamini-Hochberg [11] proposed a less conservative approach that controls the proportion of false discoveries among the overall discoveries (rejection of null hypothesis) made. These univariate statistical methods are incapable considering the highly correlated structure of metabolomics data beforehand, thus increasing the probability of obtaining false positives and false negatives.

Recently machine learning methods [12–18] have been shown as important tools to identify significant biomarkers. Principal component analysis (PCA) [19], hierarchical clustering analysis (HCA) [20, 21], self-organizing maps (SOMs) [22, 23], partial least square-discriminant analysis (PLS-DA) [19] and Random Forest [24] are widely used multivariate machine learning methods in metabolomics study. Recently, Mendez [13] used a single hidden layer artificial neural network (ANN) to discover significant metabolites and hypothesized this approach was equivalent to PLS-DA. The advantage of these multivariate methods is that they can consider all the features simultaneously and, consequently, deal with the correlation among the metabolites. However, most of these methods do not have the inherent ability to compute valid *p*-values, thus are not able to guarantee the statistical significance for the selected features. Computation of *p*-value requires the knowledge of the distribution under the null, which is generally unknown and is highly dependent on the feature selection algorithm. Post selection inference techniques [25–27] can compute valid *p*-values for the chosen features after deciding upon a model but are only applicable in restricted settings.

Barber, Candes and authors [28, 29] introduced a seminal feature selection approach called “knockoff filtering” which has the capability of handling more general model selection approaches with provable control over false discovery rate (FDR). The basic idea behind knockoff filtering is to create dummy features that are conditionally independent of the responses and satisfy pairwise exchangeability with the original features. One then concatenates the original features and these dummy features called “knockoffs” and employ any regression and classification algorithm [24, 30] to generate feature importance scores. Barber [28] proposed a new statistic called “knockoff adjusted score” by comparing the original feature importance score and corresponding dummy feature importance score. Given FDR level, a data driven threshold is then generated based on these knockoff scores for the rejection of null hypothesis with provable FDR control. One of the drawbacks of this method is that it introduces randomness in the process of generating dummy variables that may lead to high variability in the outcome. To address this problem, Nguyen [31] proposed a technique called an “Aggregation of multiple Knockoffs” (AKO). This method generates multiple copies of knockoff features independently, produces an intermediate *p*-value from the knockoff adjusted score [28] for each feature across all copies and then performs quantile aggregation on the *p*-values. AKO selects significant features by applying Benjamini-Hochberg (BHq) [11] step up procedure on the quantile aggregated *p*-values.

In this paper, we validate the use of aggregate knockoff filtering [31] for metabolomics, in particular untargeted metabolomics. We used a publicly available dataset “Longitudinal Metabolomics of the Human Microbiome in Inflammatory Bowel Disease” [32]. The study was involved in the NIH Integrative Human Microbiome Project, in which 546 samples were analyzed utilizing four different chromatographic methods to completely profile the metabolome of each sample. The methods relied on high resolution/accurate mass methods of acquisition and 597 features were annotated with confirmation by standard. We will demonstrate that aggregate knockoff filtering discovers additional biomarkers of interest, specifically when non-IBD versus CD were compared with existing methods while ensuring the control over false discovery rate. We will consider the percentage of missing values as a threshold to remove metabolites in the preprocessing step as a hyperparameter and find the best threshold that guarantees the maximum discovery of significant biomarkers related to IBD.

## Materials and methods

### Dataset

The data is available at the NIH Common Fund’s National Metabolomics Data Repository (NMDR) website, the Metabolomics Workbench https://www.metabolomicsworkbench.org under the Project ID: PR000639. The data can be accessed directly via the project DOI: 10.21228/M82T15. This work is supported by NIH grant, U2C-DK119886. Study design, instrumental methods, equipment, collection, sample preparation and other relevant study data are located within the reference cited. Of note was the comprehensive chromatography analysis utilizing four different conditions e.g. C18 Reverse-Phase negative mode acquisition, C8 Reverse-Phase positive mode acquisition, Hydrophilic interaction chromatography (HILIC) negative mode acquisition, HILIC positive mode acquisition. 546 samples were collected under each mode of chromatography condition but the number of named metabolites varies (Table 1).

**Table 1:** Number of named metabolites collected under different modes.

Mode	C18 Negative	C8 Positive	HILIC Negative	HILIC Positive
Named metabolites	91	213	115	177

### Data preprocessing

**Fig 1:**
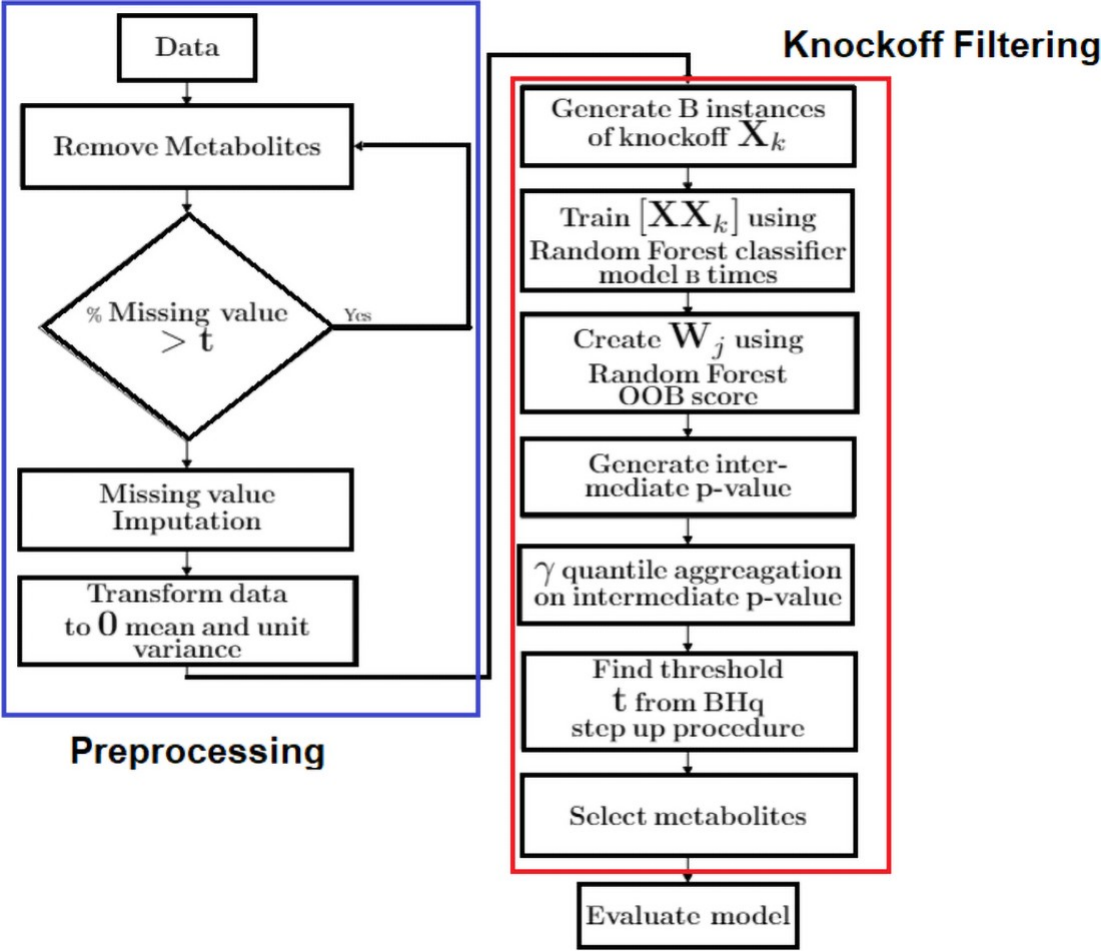
Workflow of the whole process. Blue box represents the data preprocessing step whereas blue box denotes the steps of knockoff filtering.

Several preprocessing steps were applied to each of the four datasets. Missing values are common in mass-spectrometry (MS) based metabolomics data. Since too many missing entries will cause difficulties for subsequent analysis, handling missing values is important. To address this problem, we first applied a threshold-based prefilter on each dataset to keep or remove a particular metabolite based on this threshold. In general, we kept only those metabolites that have nonzero value in at least *t*% of the total number of samples. This procedure is widely known as *t*% rule [33]. We picked threshold *t* from the set *T* = {0, 60, 70, 80, 100} that includes two extreme values 0, 100. In case of *t* = 0 we did not apply any thresholding to remove metabolites from the data. On the other hand, for *t* = 100, metabolites having at least one missing value are removed. Though these two extreme thresholds are not widely used as standards to deal with moderately large metabolomics dataset, we studied these extreme cases in order to assess the effect of this preprocessing step on the performance of our proposed method. We then apply K- Nearest Neighbor (KNN) missing value imputation [34] technique, that works based on the principle described in [35]. It is worth mentioning that for *t* = 100, missing value imputation step was not required. Before applying the knockoff filtering, the imputed data was standardized to zero mean and unit variance. Going forward we will define t as the missing value imputation threshold. The detailed workflow can be seen in Fig 1.

### Aggregate knockoff filtering on preprocessed data

We applied Aggregate Knockoff (AKO) filtering [31] on the preprocessed data. The filtering process begins by generating the knockoff of the original data matrix $\text{X}\in R^{n\times p}$, where *n* and *p* represent the number of samples and number of metabolites respectively. Knockoff data ${\text{X}}_{K}\in R^{n\times p}$ are generated by sampling from the conditional distribution $X_{K}|X∼N\left(\mu ,\text{V}\right)$ without looking at the response vector $y\in R^{n}$. We approximated *μ* (mean) and **V** (covariance) using the regression formulas stated in [29] assuming original data distribution is Gaussian. As mentioned in [31], we generated *B* instances of Knockoff copies ${\left\{{\text{X}}_{K}^{b}\right\}}_{b=1}^{B}$ independently. We set *B* = 15. Each knockoff ${\text{X}}_{K}^{b}$ and original data matrix **X** were columnwise concatenated into an augmented data $\left[\text{X}{\text{X}}_{K}^{b}\right]\in R^{n\times 2p}$ having twice the number of features compared to the original data matrix. Note that each dataset has three different classes e.g., CD, UC, non-IBD and response variable *y*_*i*_ is assigned to either 0, 1, or 2 based on the group each sample belongs to for *i* = 1, …, *n*. We applied Random Forest classifier [12] on the augmented data to generate feature importance scores. We set the number of features that are randomly selected at each node to the square root of the number of input features. Another hyperparameter for the random forest algorithm, the number of trees was set to 1000, which we obtained using cross validation. We used the absolute mean decrease of accuracy in Out-Of-Bag (OOB) samples with random permutation of features as feature importance *Z*_*j*_ for *j* = 1, …, 2*p*. OOB score is defined as the impact of each feature on the classification accuracy when removed from the input data during training. We generated the knockoff adjusted scores *W*_*j*_ by taking the difference between the absolute of the original feature importance score and absolute of corresponding knockoff feature importance score for *j* = 1, …, *p*. A large positive *W*_*j*_ ensures that variable *j* truly belongs to the model. We created an intermediate *p*- value *π*_*j*_ as defined in AKO [31] for *j* = 1, …, *p* from the knockoff adjusted score. In brief, for *B* independent draws of knockoff variables we obtained the corresponding *B* sets of knockoff adjusted score, from which we computed *p* values $\pi_{j}^{\left(b\right)}$, for all *j* = 1, …, *p* and *b* = 1, …, *B*. Then we performed *γ*-quantile aggregation introduced in [36] for each variable in parallel to get a new statistic ${\bar{\pi }}_{j}$ for *j* = 1, …, *p*. We chose *γ* to 0.5. After obtaining a list of *p*-values, we followed Benjamini-Hochberg step-up procedure [11] to select significant features given an FDR control level *α* = 0.05. (We refer the reader to S1 Appendix and references therein for detailed Knockoff filtering method).

## Results and discussion

**Fig 2:**
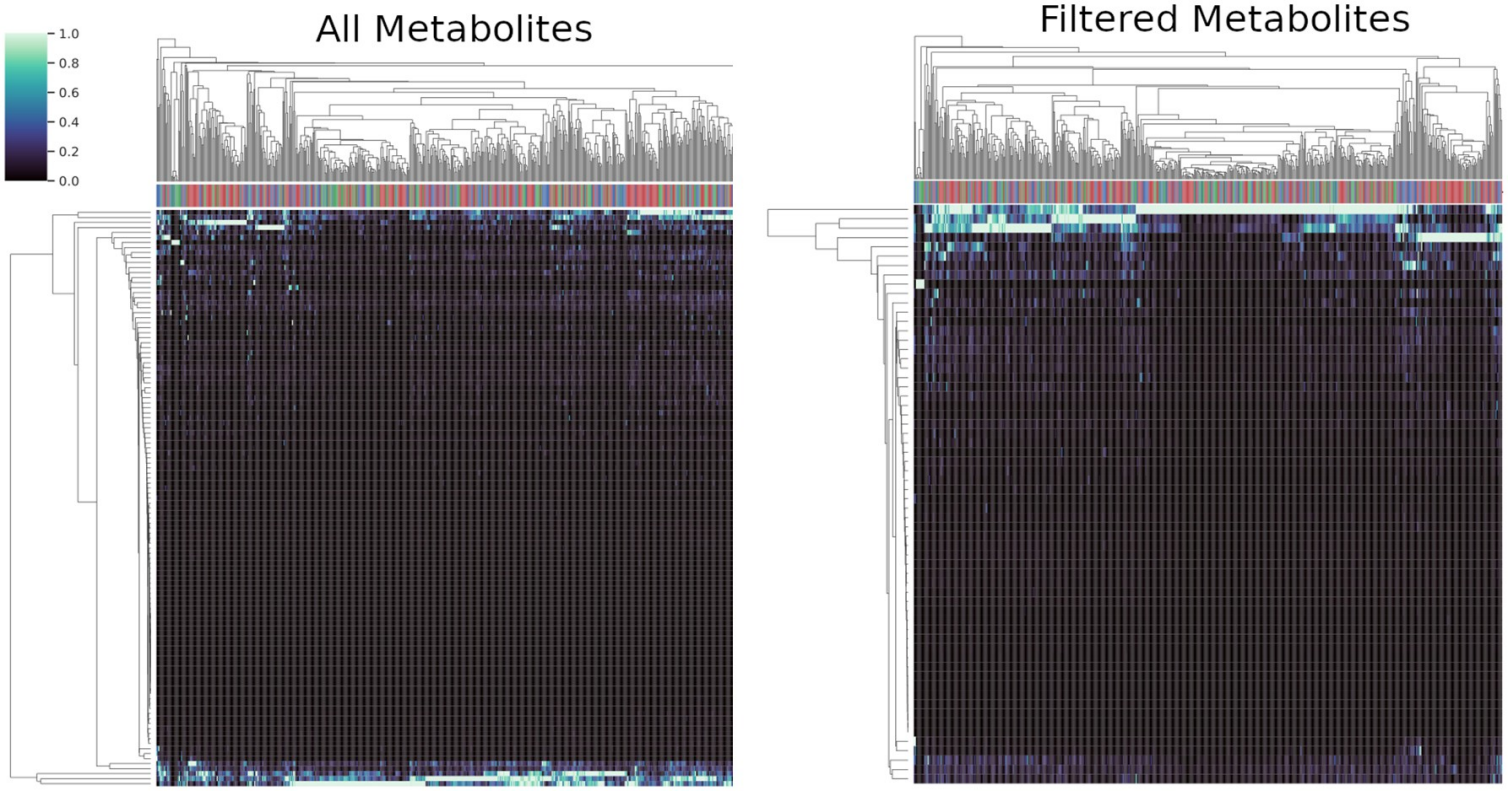
Clustermaps of the metabolites identified in the HILIC Positive acquisition group. All identified metabolites (left) vs filtering insignificantly changing metabolites with respect to sample group (right) are clustered respectively by Euclidean distance of expression levels (Each individual metabolite expression profile normalized to 1 across individuals) (x-axis) and colored by disease factor (Red for CD, Green for UC and Blue for non-IBD).

Clustering of metabolite expression levels (Fig 2) performs well in differentiating families of metabolites. This level of analysis observes CD, UC, and non-IBD samples and groups them by the expression level and trends (i.e., up- or down regulation) through the study. Due to the richness of the results from untargeted metabolomics studies, it is not feasible to look at all of the metabolites and derive scientifically accurate conclusions. A further simplification of datasets often utilize a p-value cutoff. By which, this probability calculation evaluates the occurrence of extreme results and their likelihood of reoccurrence of extreme results in support of a null hypothesis. Many researchers utilize this p-value cutoff technique, to reduce datasets to more probable groupings, resulting in more manageable datasets. While compounds of similar metabolite families group well along the right y-axis (e.g., fatty acid type molecules), overall no truly observable trends or groupings allow for greater differentiation potential concerning the non-IBD and CD samples. Unfortunately, this method often does not distill the information to a manageable level. It should also be noted that when an additional p-value cutoff is applied to the data from Fig 2, certain cholates have been dropped. The loss of those compounds is irrelevant because levels of cholates have been shown to be significantly decreased in patients with IBD compared to non-IBD patients [38]. P-value cutoffs can lead to incorrect biomarker identification, wasted computational expenses and expertise. This shows the necessity of using methods to narrow the researchers’ focus to fewer metabolites quickly, for initial review.

By utilizing the Aggregate Knockoff filtering technique, we can enrich the results by extracting out metabolites that truly are significant. We have identified different numbers of metabolites as significant based on selected thresholds for keeping metabolites in accordance with the *t*% rule (Table 2).

**Table 2:** Each cell in the table represents the number of selected metabolites under different modes of data collection and threshold.

Threshold (*t*%)	C18 negative	C8 positive	HILIC negative	HILIC positive
0%	20	9	21	23
60%	33	4	32	3
70%	35	6	28	12
80%	38	6	35	13
100%	23	3	23	27

**Fig 3:**
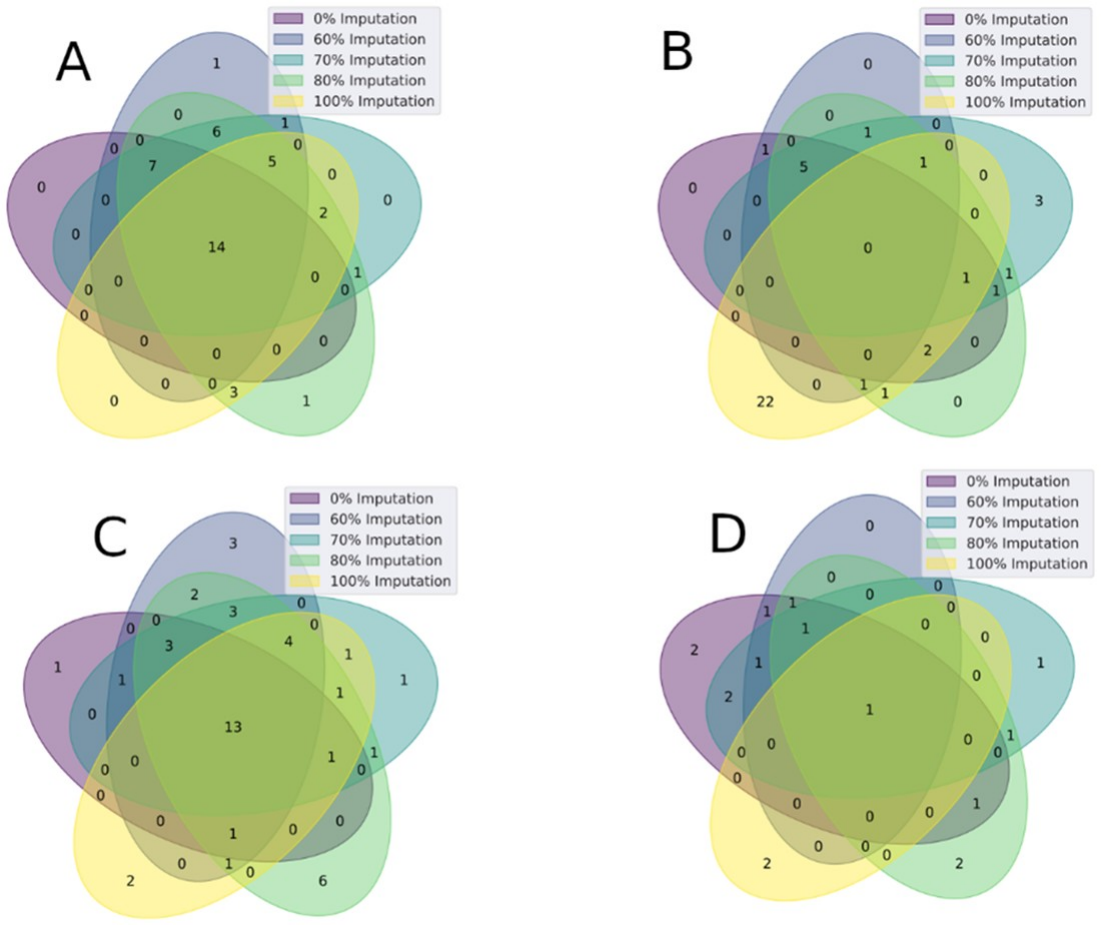
Venn overlaps of the metabolites identified by knockoff filtering of the C18 Negative (A), HILIC Positive (B), HILIC Negative (C) and the C8 Positive (D) chromatography groups.

The maximum number of metabolites discovered was achieved at various missing value imputation thresholds for different datasets. The reason behind obtaining different thresholds for different dataset is that each data was collected under a certain condition. Therefore, the quality of the data varies so as the missing value percentage. As an example, the maximum number of metabolites for the C18 negative dataset is observed when the threshold is 80%; however, for the HILIC positive data, the threshold is 100%. The missing value imputation level appeared to have an effect on which metabolites are selected as of interest, therefore in order to obtain the largest coverage of metabolites selected by the knockoff filtering methodology, different missing value imputation levels (0%, 60%, 70%, 80%and100%) were employed and results aggregated under each respective chromatography group (Table 2). The knockoff filtering appears to improve with smaller initial metabolite groups (HILIC Negative = 115 Metabolites, C18 Negative = 91 Metabolites vs. HILIC Positive = 177, C8 Positive = 213). C18 Negative had 15 metabolites consistently identified among each missing value imputation level but optimal coverage at 80% (Fig 3). This was not consistent in the HILIC Positive group as one missing value imputation level (100%) contains a large majority of unique metabolites vs the other levels. This shows the need to leverage various missing value imputation levels to not exclude potentially important metabolites.

**Fig 4:**
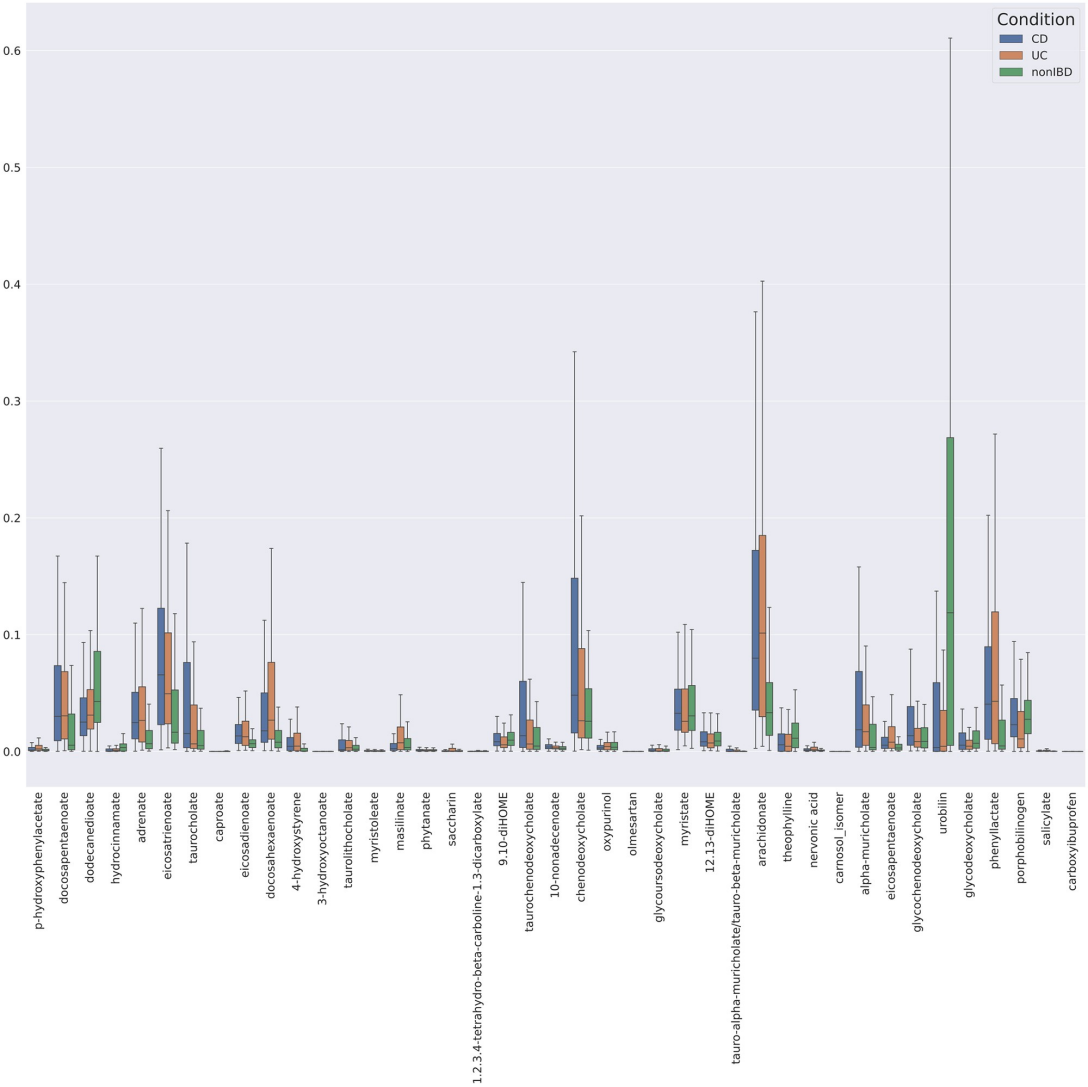
Boxplot of selected metabolites from C18 negative dataset.

There are noteworthy metabolites identified that have been shown in literature to be affected in IBD populations in the C18 chromatography group (Fig 4). Arachidonate (arachidonic acid) has been shown dysregulated in IBD patients, with decreasing fold change [32]. While not a direct essential fatty acid, there is some debate regarding linoleic acid and its conversion to arachidonate to account for a deficiency in the aforementioned [37]. The Bacteria-Protease-Mucus-Barrier hypothesis suspects that saccharin may dysregulate gut bacteria and inactivate key digestive proteases [38]. Docosapentaenoate was found to be downregulated in patients [39]. Eicosatrienoate (eicosatrienoicac acid) showed dysregulation in Crohn’s Disease [40]. There were 212 detected enrichments in cholate bile acids, including glycine and taurine conjugates [32, 41]. Numerous salicylates have been associated with IBD, including oral dosing for prevention of IBD relapse [42, 43]. Other fatty acids, like eicosadienoate, have been tracked as a possible biomarker [44]. Comprehensively, many of the other metabolites listed are also found in literature, however numerous have been tracked in one study [32]. “S2 Table” details the metabolites that were identified by running each of the datasets that were identified by running each of the datasets through aggregate knockoff filtering.

**Fig 5:**
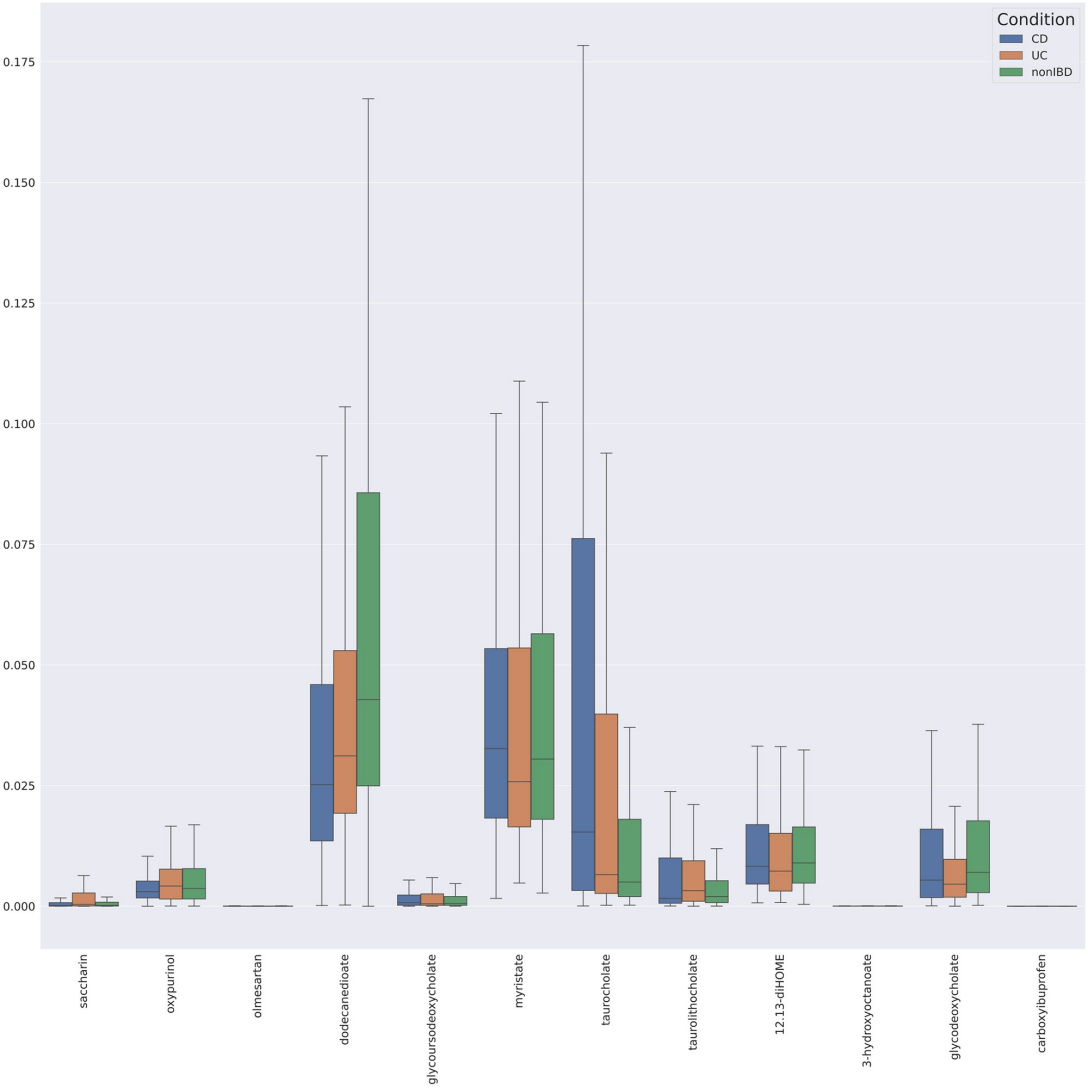
Boxplot of metabolites identified as important by knockoff filtering but do not pass a p-value filter. CD expression shown in blue, UC in Orange and non-IBD in Green.

The Knockoff filtering method can also extract metabolites that do not pass the *p*-value threshold (Fig 5). These identified metabolites have also been associated with IBD-focused research. Elevated concentration of 12,13-diHOME (12,13-dihydroxy-9Z-octadecenoic acid) impedes immune tolerance in fecal material [45]. Additional cholates were identified from this knockoff filtering method, including additional taurine conjugates [32]. Carboxylates, such as 1,2,3,4-tetrahydro-beta-carboline-1,3-dicarboxylate, were elevated, and these metabolites significantly correlate with disease prediction [46]. Dodecanedioate was also identified as important [47]. With respect to UC subjects, the model revealed variations in the occurrence of dicarboxylic acids, such as undecanedioate, dodecanedioate and sebacate, which are proposed to regulate mitochondrial fatty acid oxidation and to be involved in IBD-related liver dysfunctions.

**Fig 6:**
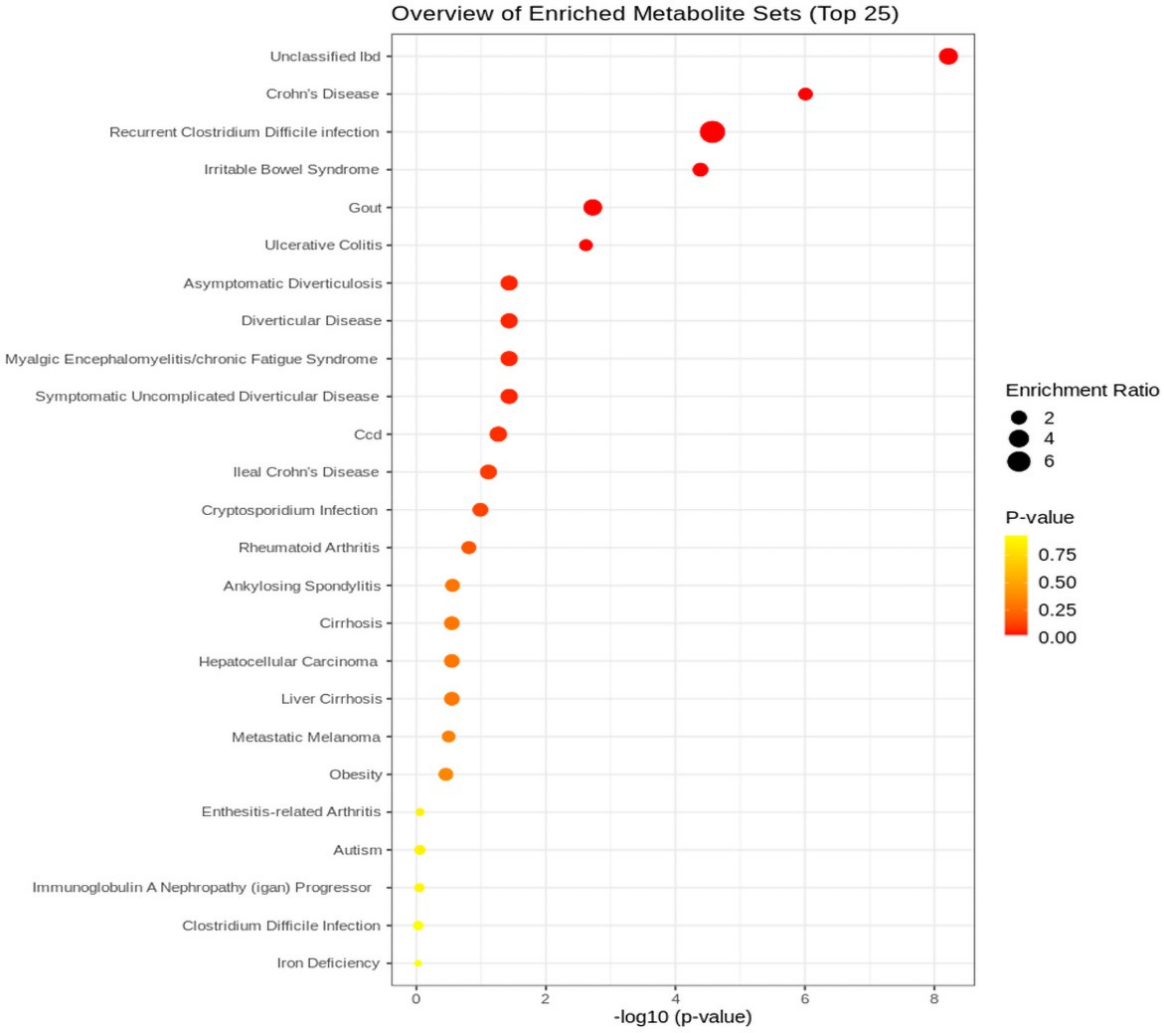
Metaboanalyst enrichment mapping of unique features identified by algorithm.

Upon performing an enrichment analysis on the metabolites uniquely identified by the algorithm for disease signature utilizing Metaboanalyst [48]. Unclassified IBD, Ulcerative Colitis, and Crohn’s Disease are all significantly enriched (Fig 6). This provides a secondary validation that the metabolites selected from the algorithm have a role in IBD.

Knockoff filtering is shown to be a great tool to discover important metabolites that were not identified by the p-value cutoff method, which is widely used in many existing metabolomics processing tools e.g., Metaboanalyst. However, the performance of the knockoff filtering method is highly dependent on the variable selection algorithms that are used to generate feature importance scores which, in turn is sensitive to data preprocessing steps e.g., pre-filtering, missing value imputation technique. One of the drawbacks of the proposed method is that we generated the second-order knockoffs by approximating mean and covariance assuming data distribution is Gaussian. In cases where this assumption is not satisfied, the proposed method will generate poor quality knockoffs and consequently lead to poor performance. This knockoff method also suffers from false discovery vs. power tradeoff like all the existing works that only control the FDR. Future study can be conducted with recently developed generative model-based knockoff generation techniques [49–51], as well as in the direction of increasing the power of detection while keeping the FDR below a significant level.

## Conclusion

Utilizing knockoff filtering in combination with more traditional techniques (i.e., p-value cutoff) improves researchers’ abilities to sift through the large amounts of data that are generated in metabolomic experiments. The combination of aggregate knockoff filtering and p-value cutoffs allows for more rapid secondary validation and additional hypothesis generation than taking the time tracing down the dead-end leads. Aggregate Knockoff filtering technique also produces metabolites that simple p-value filtering misses. These metabolites have been implicated in having a roll in CD/IBD and would otherwise go unseen if not for the Knockoff filtering method. Aggregate knockoff filtering method also ensures the statistical significance of the selected metabolites which may not be guaranteed in case of many traditional machine learning techniques. In conclusion, this paper introduces the knockoff filtering technique to the metabolomics community which is shown to be a better tool to identify metabolites with statistical guarantee.

## Supporting information

S1 Fig Box plot of the metabolites in the C8 positive, HILIC negative and HILIC positive. Contains metabolites that are selected by the knockoff filtering algorithm but do not pass a *p*-value filter of 0.05 for three different datasets.
(PNG)

S1 Table Identified metabolites from the knockoff filtering methodology utilizing aggregate repeated missing value imputation. This table contains the union of metabolites coming from the sets of identified metabolites using different thresholds for each dataset.
(XLSX)

S2 Table Metaboanalyst output of an enrichment analysis of the metabolites that were identified by the algorithm but are not identified by a p-value selection. This table contains enrichment of the metabolites that only passed through the knockoff filtering.
(XLSX)

S1 Appendix Supplementary notes. In the notes we provide some background theory on Model-X Knockoff filtering and Aggregate knockoff filtering method.
(TEX)

S1 Code Python code. (TXT)
