# Supplementary figures and images for: Utilizing machine learning with knockoff filtering to extract significant metabolites in Crohn’s disease with a publicly available untargeted metabolomics dataset
Source: PLoS One. 2021 Jul 29;16(7):e0255240. doi: 10.1371/journal.pone.0255240 (PMC8320926; doi:10.1371/journal.pone.0255240)

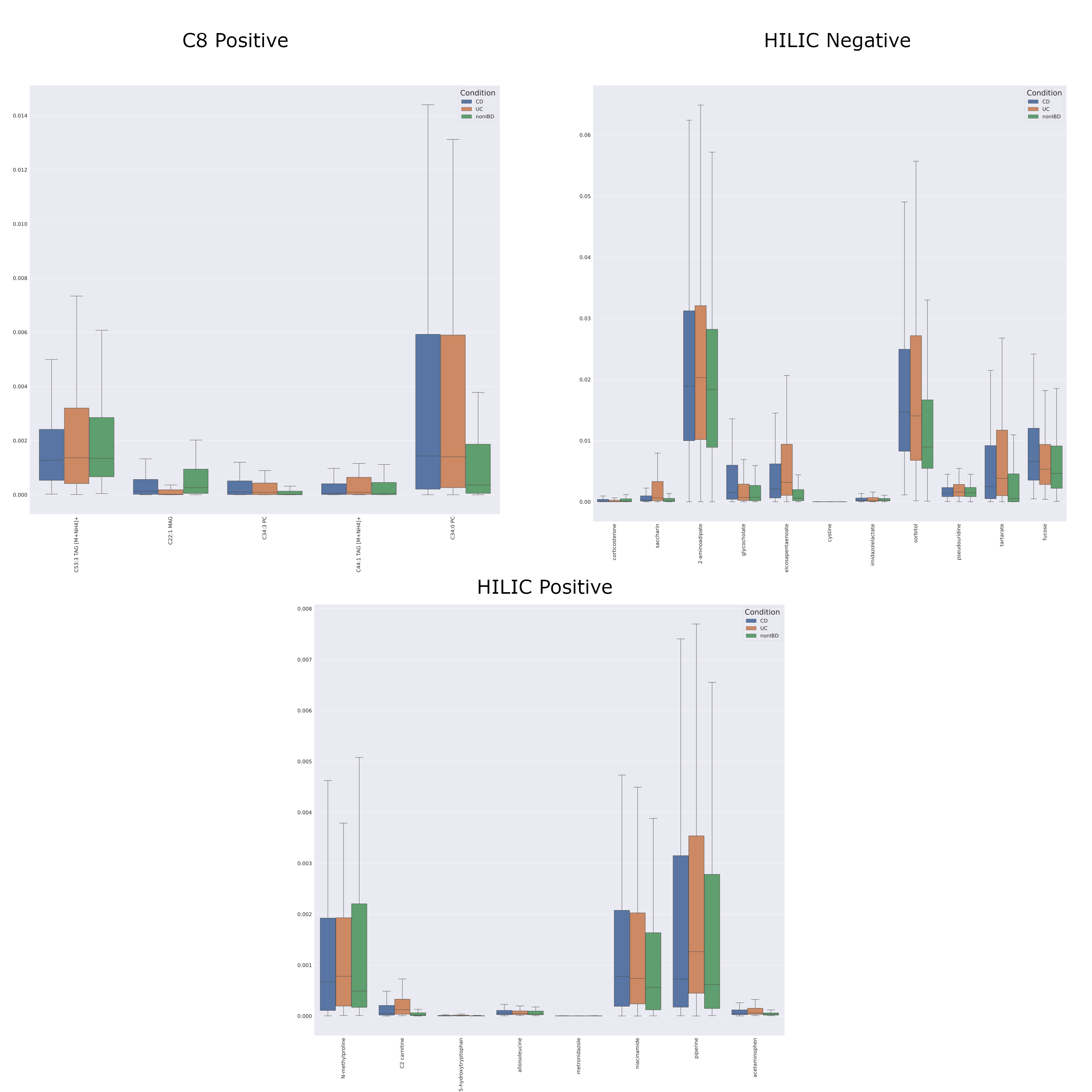

Supplement: S1 Fig — Contains metabolites that are selected by the knockoff filtering algorithm but do not pass a p-value filter of 0.05 for three different datasets. (PNG) [file pone.0255240.s001.png]
